# Protein Dynamics in the Plant Extracellular Space

**DOI:** 10.3390/proteomes4030022

**Published:** 2016-07-13

**Authors:** Leonor Guerra-Guimarães, Carla Pinheiro, Inês Chaves, Danielle R. Barros, Cândido P. Ricardo

**Affiliations:** 1 Centro de Investigação das Ferrugens do Cafeeiro (CIFC), Instituto Superior de Agronomia (ISA), Universidade de Lisboa (UL), Quinta do Marquês, 2784-505 Oeiras, Portugal; 2 Linking Landscape, Environment, Agriculture and Food (LEAF), Instituto Superior de Agronomia (ISA), Universidade de Lisboa (UL), 1349-017 Lisboa, Portugal; 3 Instituto de Tecnologia Química e Biológica (ITQB), Universidade NOVA de Lisboa (UNL), 2780-157 Oeiras, Portugal; cm.pinheiro@fct.unl.pt (C.P.); ichaves@itqb.unl.pt (I.C.); ricardo@itqb.unl.pt (C.P.R.); 4 Faculdade de Ciências e Tecnologia, Universidade NOVA de Lisboa (UNL), 2829-516 Caparica, Portugal; 5 Instituto de Biologia Experimental e Tecnológica (IBET), 2780-157 Oeiras, Portugal; 6 Departamento de Fitossanidade, Faculdade de Agronomia Eliseu Maciel (FAEM), Universidade Federal de Pelotas (UFPEL), 96010-610 Pelotas-RS, Brazil; danrbarros@hotmail.com

**Keywords:** apoplastic fluid, biological processes, cell wall, dicot plants, intercellular space, proteomic analysis

## Abstract

The extracellular space (ECS or apoplast) is the plant cell compartment external to the plasma membrane, which includes the cell walls, the intercellular space and the apoplastic fluid (APF). The present review is focused on APF proteomics papers and intends to draw information on the metabolic processes occurring in the ECS under abiotic and biotic stresses, as well as under non-challenged conditions. The large majority of the proteins detected are involved in “cell wall organization and biogenesis”, “response to stimulus” and “protein metabolism”. It becomes apparent that some proteins are always detected, irrespective of the experimental conditions, although with different relative contribution. This fact suggests that non-challenged plants have intrinsic constitutive metabolic processes of stress/defense in the ECS. In addition to the multiple functions ascribed to the ECS proteins, should be considered the interactions established between themselves and with the plasma membrane and its components. These interactions are crucial in connecting exterior and interior of the cell, and even simple protein actions in the ECS can have profound effects on plant performance. The proteins of the ECS are permanently contributing to the high dynamic nature of this plant compartment, which seems fundamental to plant development and adaptation to the environmental conditions.

## 1. Introduction

### 1.1. The Extracellular Space or Apoplast

**Figure 1:**
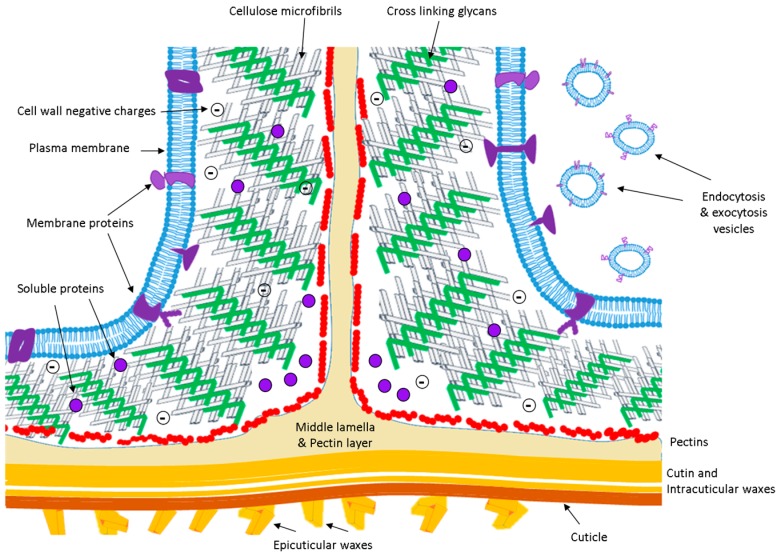
A schematic representation of the extracellular space of an angiosperm (not at scale). Main components: plasma membrane (blue); crosslinking glycans (green); cellulose microfibrils (grey); middle lamella and pectin layer (beige); pectins (red); and membrane and soluble proteins (violet). In Poaceae, pectins are only present in the middle lamella.

The pioneering microscopic observation of *Quercus suber* cork by Robert Hooke’s in the 1660s paved the way to the evidence that plant cells are encased in an exoskeleton-like structure that confers shape, stability, and protection, and unites the cells in the tissues. What was initially considered a kind of “wood box” is now known to be a cellular compartment that plays important roles in absorption and transport of solutes, defense, intercellular communication, metabolic regulation, environmental sensing, and growth. Extracellular space (ECS) and apoplast are often used as synonyms and with the broadest meaning, i.e., referring to the whole compartment external to the plant plasma membrane (Figure 1), that includes the cell wall, the free space between cells (or intercellular space), and a fluid (apoplastic fluid; APF). Since the apoplast is envisioned as the free diffusion space located outside the plasma membrane it can be considered a continuous system in the whole plant, extending from the roots to the leaves and including the dead cells of xylem, but interrupted by the Casparian strip in roots.

### 1.2. The Cell Wall

The major structural component of the extracellular space is the cell wall, which has a complex composition varying between tissues, the cell differentiation state, the type of the plant, and the influences of environment. Five types of polymers are the main components: cellulose, crosslinking glycans (or hemicellulosic polysaccharides), pectins, proteins, and lignin (that gives rigidity and strength to the secondary wall). A model for the primary wall during cell growth was provided by Carpita and Gibeaut [1] and Somerville et al. [2] which discussed the importance of making use of a system-based approach to understand plant cell walls. In 2010, Plant Physiology dedicated a special issue to the analysis of the plant cell wall aiming to elucidate “*the structure and functional properties of plant cell walls which remains a daunting scientific challenge*” [3]. It is now evident that the cell wall composition reflects plant evolutionary relationships [4].

The established network of the cell wall components confers a given porosity to the matrix, i.e., the three-dimensional space in which molecules can diffuse. While water and ions can freely move in the extracellular matrix, it is considered that the movement of particles with a diameter greater than ≈4 nm is conditioned [5]. Roughly, proteins as small as 20 kDa are considered too large to move freely within the network.

The hydroxyproline-rich glycoproteins (HRGPs), or extensins, were the first to be discovered (in the 1960s) followed by proline-rich proteins (PRPs), glycine-rich proteins (GRPs) [6,7], and arabinogalactan-proteins (AGPs) [8,9], which belong to the HRGP family. It was, thus, evident that the cell wall was composed of two structurally independent but interacting networks: the polysaccharides and the structural proteins. The structural proteins are strongly bound in the wall due to covalent bonds, remaining attached even after salt extraction [10]. Cell wall structural proteins are a small fraction of the total extracellular proteins so far detected. Indeed, of the 281 extracellular proteins present in databases in 2005, Jamet et al. [10] referred that only 1.5% of extracellular proteins were structural proteins. The crosslinking of the structural proteins to the wall polysaccharides is a dynamic process in the cell, developmentally controlled and influenced by the environment. Thus, its alteration affects the wall rigidity and resistance to degradation, with marked implications on the properties and behavior of the wall, extending from cell elongation to many developmental processes and abiotic and biotic plant responses [6,11].

### 1.3. The Proteins of the APF

Soluble proteins of the ECS can be of two types, considering their interactions: (1) proteins with little or no interactions with the wall components, freely soluble in the ECS, and that can be extracted with low ionic strength buffers; most of these proteins have acidic pI from 2–6; and (2) proteins weakly bound to the wall matrix through hydrogen bonds, van der Waals, hydrophobic, or ionic interactions and that can be extracted by salts; most of these proteins are basic with pI from 8–11 [12].

APF proteins are collected by a technique similar to that described in 1965 by Klement [13]. The intervein tissue of the leaf lamina is cut lengthwise into strips about 1.5 cm wide, vacuum-infiltrated with distilled water or a buffer solution, and then centrifuged at low speed to collect the APF. Rathmell and Sequeira [14], using this technique, demonstrated the presence of peroxidase in the APF of tobacco leaves, which had little contamination from the cytoplasm. While discussing the eventual role of the extracellular peroxidase these authors refer to previous work by Ridge and Osborne [15], reporting on the presence of insoluble forms of peroxidase, covalently and ionically bound to pea cell walls. The vacuum-infiltration techniques have been largely utilized to identify APF proteins, mainly from leaves but also from other organs, for instance, roots and stems [16,17,18,19,20,21]. The protein yield of the vacuum-infiltration technique is generally low and, despite the use of infiltration buffers of increased molar strength, some proteins resist solubilization due to their ionic interactions with wall components. So, harsher methodologies have to be applied with the eventual inconvenience of getting higher levels of cytoplasmic contamination [22]. Non-disruptive methods have also been used to reduce contamination, such as the analysis of the growth medium of cell cultures. This method allows the study of secreted proteins but has limited biological significance.

Extracellular proteins, that should have a signal peptide, are secreted by the endomembrane system formed by the endoplasmic reticulum, the Golgi system, and the secretory vesicles that constitute the exocytosis pathway [23]. However, some proteins collected in APFs lack the signal peptide and are not thought to be contaminants from other cell compartments but “non-canonical secreted proteins” or leaderless secreted proteins [19,24]. In yeast and animals cells, several unconventional transport pathways for protein secretion have been reported, but these have not been described for plants [24,25]. For plant cells, secretome proteomics show that a high proportion of proteins in the ECS do not display the canonical signal peptide [26]. Bioinformatic tools have been used to confirm the apoplastic nature of these proteins, and exciting data about new routes of secretion and a new organelle (exocyst-positive organelle—EXPO) are emerging [27].

The complexity of the ECS is reflected in the high number of different proteins found (up to 2000) and their distribution within several families (circa 30) [10,20,28]. Several protein families have been shown to act together in ECS events, but a global picture is still missing [29].

### 1.4. Proteins of the Plasma Membrane Related with the ECS

Many proteins of the plasma membrane have marked influence in the properties or functioning of the apoplast. Some participate in the synthesis of apoplastic components as is the case of the rosette complexes responsible for the synthesis of cellulose [30]. Another important group that interacts with the apoplast is that of the GAPs (glycosylphosphatidylinositol (GPI)-anchored proteins) that are covalently attached to GPIs embedded in the lipid layer of the external surface of the plasma membrane. They belong to several families (e.g., proteases, glycosyl hydrolases) and are implicated in growth and developmental processes, extracellular matrix remodeling and signaling mechanisms [31,32]. GAPs can be glycosylated with arabinogalactan (AG) chains and are, thus, AGPs. In Arabidopsis, up to 40% of GAPs can be AGPs [8,9]. The GPI anchor can be cleaved releasing the AGPs to remain free in the apoplastic fluid or be attached to cell wall components. Some AGPs, while GPI-anchored to the plasma membrane, are also covalently bound to the cell wall and so establish a physical link between membrane and wall [33]. Mutant analysis in Arabidopsis show that GPI-anchored proteins are important in cell wall reorganization, acting for instance, on a pathway that regulates cellulose synthesis in roots [30,34].

An additional and large group of transmembrane proteins found in plant genomes are the receptor-like kinases (RLKs) [35]. Examples are: wall-associated kinases (WAKs), lectin receptor kinases (LecRKs), and receptor kinases containing Leucine-rich repeats (LRRs). These proteins have a cytoplasmic kinase domain and extracellular specific domains, and it was proposed they function by sensing changes in the cell wall structure and transduce the message to the cytoplasm [33].

### 1.5. A Proteomics Approach to the ECS

The increasing knowledge on the ECS has reinforced the view of the fundamental role played by this compartment for cell function and plant metabolism. Information on the ECS proteins was initially based on classical biochemical techniques subsequently greatly improved through the use of proteomic methodologies. Two-dimensional gel electrophoresis (2-DE) is the most common technique used for protein separation and mass spectrometry (MS) for their identification. The 2-DE delivers a map of total protein fraction that reflects changes in protein expression level, isoforms, or post-translational modifications (PTM). The successful identification of the ECS proteins is dependent on extraction and separation, amenability to MS analysis, and protein annotation in the sequenced databases. The analysis of the proteome is, therefore, a key method to provide systems-level information about protein function in time and space, and to obtain a concise view of biological processes. The present article aims to compile an update of the existing knowledge on ECS proteins using a proteomic approach.

## 2. Bibliographic Search (Description of the Paper Dataset of the Review)

**Figure 2:**
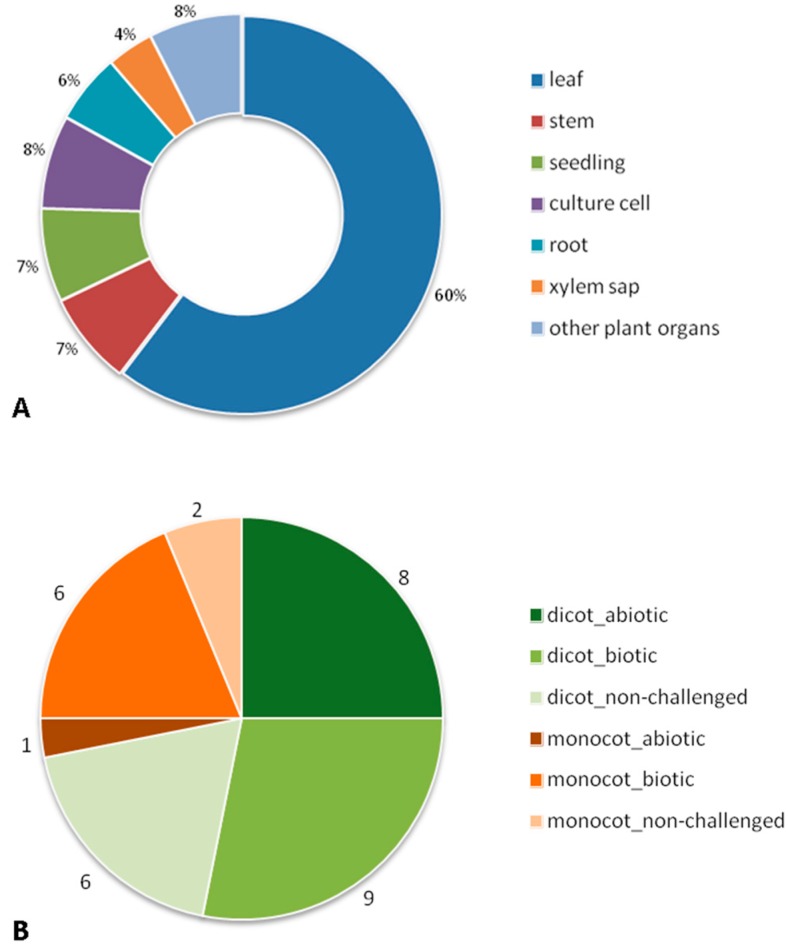
Graphical representation of the 52 original research articles considered in our review by: (**A**) plant organs; (**B**) leaf papers in the three experimental conditions (abiotic and biotic stress, and non-challenged) of dicotyledoneae and commelinid monocot plants.

A bibliographic search on the Web of Knowledge (http://apps.webofknowledge.com, 1 December 2015) was made using as search topic “plant apoplastic proteom*”. This survey identified 83 papers that used proteomics methodologies for the study of ECS proteins of *Angiospermae*. When considering only peer-reviewed original research articles, this number was reduced to 52 papers (Supplementary Table S1). Five out of the 52 papers integrated other –omic methodologies: transcriptomics (two papers; [36,37]) and metabolomics (three papers; [38,39,40]). More than half of these papers are on leaf (60%), followed by stem (7%), seedlings (7%), and root (6%) (Figure 2A). Although xylem is an apoplast component, only two papers, both on *Brassica oleracea,* specifically consider it [38,41]. The papers we analyzed date from 2003 and the most popular method for protein fractionation used was 2-DE (39 out of 52). Differential in-gel electrophoresis (DIGE) was used in six papers. Starting in 2009, some papers combine both gel-based (1D) and gel-free methodologies for plants with sequenced genomes. When different conditions of stress (abiotic and biotic) and non-challenged were considered, the lack of proteomic data on different organs led us to focus on leaves. The taxonomy of our dataset (leaf) shows that the commelinid monocot plants were represented by several genus of just the Poaceae family, while the dicotyledoneae (dicot) plants were represented by different genus and families: Brassicaceae, Fabaceae, Rubiaceae, Solanaceae, Actinidiaceae, Salicaceae, Amaranthaceae, Rosaceae, and Vitaceae. These leaf papers can be divided by experimental conditions: nine papers on abiotic stress, 15 on biotic stress, and eight on non-challenged plants (Figure 2B). To extract the same type of information on the proteins described in the papers we faced the difficulty of the insufficient descriptions of datasets and different terminologies used by the several authors, problems already reported by Jorrin-Novo [42]. For this reason we were not able to perform a bioinformatic functional annotation of the proteins (e.g., BLAST2GO) but, instead, we did a manual inspection of the data. Consequently, when no annotation was given by an author we ascribed a biological process to the protein on the basis of the information provided in the dataset (Table S1). The major limitation of this approach was that each protein was only ascribed to one biological process, when, potentially, it could be involved in several processes.

## 3. The Biological Processes Detected in the ECS

**Figure 3:**
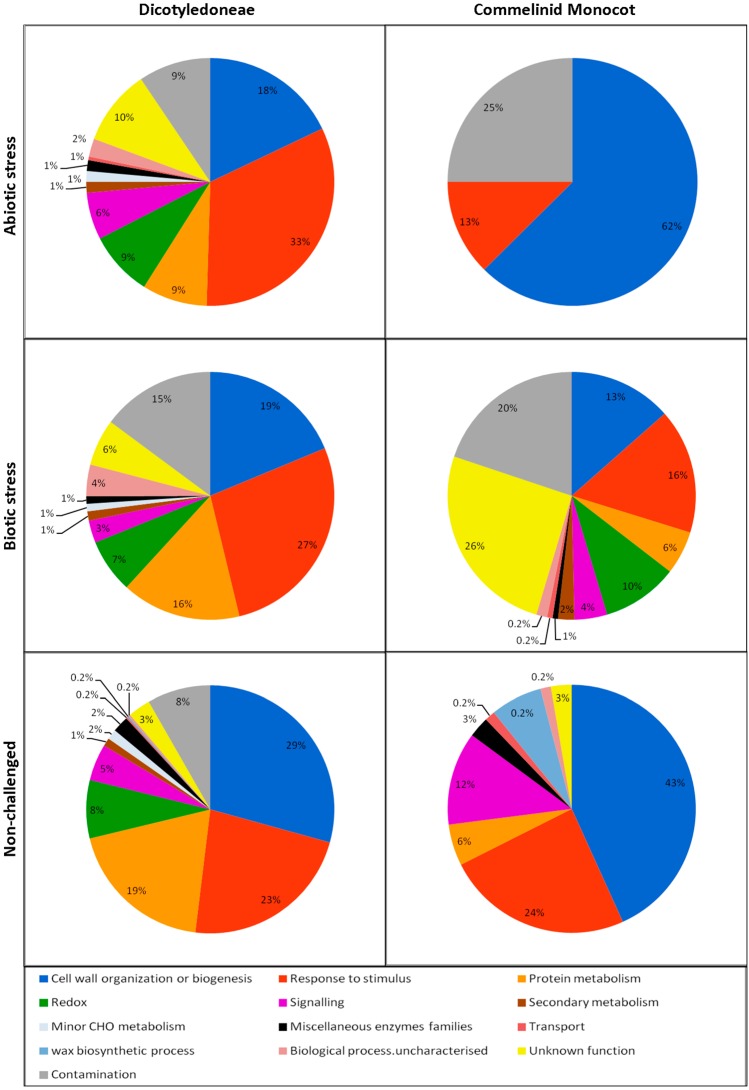
Functional categorization of the identified leaf APF proteins into biological processes in the three experimental conditions (abiotic and biotic stress, and non-challenged) of dicotyledoneae and commelinid monocot plants.

From the proteins identified in our review it is inferred that the main biological processes active in the ECS are “cell wall organization and biogenesis”, “response to stimulus” and “protein metabolism” for both commelinid monocots and dicot plants (Figure 3). However, the reduced number of papers and proteins found for commelinid monocots weakens these results and limits further considerations about this type of plant. Regarding dicots, we consider that the number of papers is sufficient to support the extraction of useful information on ECS proteins. When comparing the different experimental conditions (abiotic and biotic stress, and non-challenged plants) in the dicots we found that for both stress conditions the proteins functional categorized in “response to stimulus” were the most represented. Conversely, what is evident in the non-challenged dicots are the proteins categorized in “cell wall organization and biogenesis”. Furthermore for the stress studies it appears that in the biotic stress, alterations in the “protein metabolism” are more represented than in the abiotic stress.

### 3.1. Cell Wall Organization or Biogenesis 

Concerning the cell wall structure, the present review indicates that 18% to 29% of the total apoplastic proteins are implicated in cell wall organization and biogenesis, in the three experimental conditions (biotic and abiotic stresses, and non-challenged). This high figure implies that the cell wall undergoes appreciable modulated changes during the marked cellular events associated with those experimental conditions. This is not an unexpected result since the extracellular matrix is a complex and very dynamic structure greatly implicated in development, growth, and survival of the plant.

The majority of the proteins that changed in abundance, irrespective of the experimental condition, were glycoside hydrolases (GHs) and carbohydrate esterases (CEs) which was an indication that the processes that were occurring in the cell walls were mostly related to polysaccharide breakdown and modification [43,44], and concern the hemicelluloses and pectins of the primary wall. GHs are a numerous group of enzymes that in the Arabidopsis genome (TAIR home page) are distributed in 31 families comprising 379 members. In our analysis, the main GHs detected are β-glucosidases (GH 1 family with 48 members), β-xilosidases (GH3 family with 14 members) and β-galactosidases (GH35 family with 18 members). Also present are polygalacturonase (GH28 family with 68 members), xyloglucan-endotransglucosilase/hydrolase (XET; GH16 family with 33 members), and several α-glycosidases (acting on arabinose, fucose, galactose, glucose, and xylose). The GHs, in addition to being numerous, are also, in general, of low specificity, and so these enzymes have quite a broad spectrum of activities. This implies that they will have the capacity of acting on a diversity of cell wall polysaccharides. According to Minic et al. [43], this could be advantageous to the plant, since a small number of enzymes can achieve an efficient modification of the complex assemblage of cell wall polysaccharides. The detection of these GHs indicates that of the major hemicelluloses (xyloglucans, xylans, and mannans) the most affected are the xyloglucans which are the predominant hemicelluloses of most dicots cell wall. By interacting with the microfibrils of cellulose xyloglucans, these have the important function of forming a network that strengthens the primary cell wall [45]. In this context, the detection of XETs in the APF is very relevant. These enzymes catalyze the splitting of xyloglucan chains with the linking of the newly-generated reducing end to the non-reducing end of another xyloglucan chain, thereby, loosening the cell wall and promoting rapid wall expansion to occur [46]. The expansins that were detected are also important in this process. These proteins were discovered in association with cell wall expansion during cell growth and it was later found that α-expansins and xyloglucans interact in the process of wall loosening [47,48].

In respect to pectins, the detection of polygalacturonases (pectin hydrolases) and of rhamnogalacturonase indicates the hydrolytic transformation of pectins. The fact that CEs acting on pectins are the second most numerous group of proteins present in APF (in the experimental conditions of our review) is a further indication of intense pectin alterations in the cell walls. The Arabidopsis TAIR homepage refers five families of CEs (comprising 98 members) of which pectin esterases (CE8 and CE13 families), pectin methylesterases (CE8 family), and pectin acylesterases (CE13 family) are the most represented CEs in our analysis. These enzymes are important in altering pectin’s structure, with marked implications in the plant development and stress responses [49,50]. The detection of inhibitors of enzymes acting on pectin indicates, furthermore, the occurrence of important regulation of the transformation of cell wall pectin components. Indeed, polygalacturonase-inhibiting proteins (PGIPs) that inhibit the pectin-depolymerizing activity of polygalacturonases secreted by microbial pathogens and insects was also detected [51].

The detection of several other proteins reinforces information on the mechanisms that are taking place in the cell wall during the processes under analysis. For instance, proline-rich proteins (PRPs) are structural proteins of the cell wall that are important for its integrity [7] and that have been implicated in several plant processes, such as defense reaction to pathogen infection [52] and to drought [53]. So, our analysis reveals that major changes are occurring in the cell wall involving most of the wall components, structural polysaccharides, hemicelluloses, pectins, and glycoproteins. An important question to answer is, how distinct the reorganization of the cell wall is during the three experimental conditions? Considering the small number of publications available for the review sound statistical conclusions cannot be drawn, but main tendencies can be suggested. The same types of proteins were detected in the three experimental conditions, although it seems that in the non-challenged conditions the relative participations of proteins acting on pectin is higher than in stress situations. However, the events that are occurring in the ECS are very complex and it is notorious that many of the detected GHs and other enzymes, apart from being involved in cell wall rearrangements, can simultaneously participate in signaling, defense, and redox metabolism.

### 3.2. Response to Stimulus

The most abundant proteins included in this section are the pathogen-related proteins (PR-proteins) that represent 23%–33% of the total APF proteins. In spite of the relative differences in the three experimental conditions, the most represented proteins detected are of the same classes and are chitinases, β-1,3-glucanases, thaumatin-like proteins, germin-like proteins, PR-1, and protease inhibitors [20,36,37,39,54,55,56,57,58].

Chitinases (PR-3, 4, 8, 11) together with β-1,3-glucanases (PR-2), are well-characterized GHs (GH18 and GH17) with potential to degrade microbial cell wall components, limiting pathogen growth. The pathogen cell wall fragments (PAMP-associated molecular patterns) can function as signals contributing to resistance [20,59,60]. Leah et al. [61] and Mauch et al. [62] showed that the antifungal properties of plant chitinases are enhanced when in combination with β-1,3-glucanases, and Zhu et al. [63] reported the decrease in susceptibility in fungal attack in transgenic tobacco plants over-expressing both enzymes. The detection of chitinases in abiotic stress can be explained by the enzymatic activity on arabinogalactan as referred by van Loon et al. [64]. Chitinases and β-1,3-glucanases also constitute a storage form of nitrogen and might contribute to the maintenance of the plant organs during environmentally unfavorable periods [65]. These findings indicate that PR-type proteins can have a developmental role and, through their enzymatic activities, may generate signal molecules that could act as endogenous elicitors in morphogenesis. Other GHs with antifungal activity, well represented in the apoplast of dicot plants, are the thaumatin-like proteins (GH64; PR-5). PR-5 proteins can affect fungi, either by disrupting its membranes or by hydrolyzing β-1,3-glucans of the cell walls [66,67].

The germin-like proteins (GLPs) are a large and heterogeneous family of proteins, expressed in several plant organs and developmental stages, responding to abiotic and biotic stresses [20,56,68,69,70]. Three different enzymatic activities have been associated with these proteins: oxalate oxidase (OxO), superoxide dismutase (SOD), and ADP-glucose pyrophosphatase or phosphodiesterase (AGPPase) [69]. The PR-15 (OxO) and PR-16 (OxO-like SOD) can generate hydrogen peroxide (H_2_O_2_) and so play a dual role: creating a toxic atmosphere for pathogens and/or functioning in signaling of plant stress/defense responses [19,64]. In *Brassica napus*, it was suggested that GLPs are likely to participate in the *Sclerotiorum sclerotiorum*-induced apoplastic formation of H_2_O_2_ and may act in concert with NADPH oxidases and peroxidases, enzymes known to be involved in the apoplastic oxidative burst in response to pathogen stress [70]. The apoplastic localization of these proteins in combination with the H_2_O_2_ generating SOD activity offers a role in cell-wall fortification through the crosslinking of proteins and carbohydrates [70].

The several PR-1 proteins, despite their unknown biological functions, are often used as markers of biotic stress [20,71], but in this review they were also found in abiotic stress and the non-challenged conditions [12,19,37,72,73]. All PR-1 proteins have similar structure and are classified in the same family on the basis of sequence homology although they can have different properties and differ substantially in biological activity [64].

Some proteinase inhibitors are also considered PR-proteins (PR-6). They are induced in abscission zones and might be involved in cell wall loosening or in defense of the scarified tissue to pathogens [64]. Indeed, in our review, proteinase inhibitors represented an important class of proteins induced during biotic stress and non-challenged condition. The proteinase inhibitors that were detected are of the cysteine peptidase type (cystatin family), with essential roles in hypersensitive cell death [74,75,76] and of the serine peptidase type (Kunitz family trypsin inhibitor proteins, tumor-related proteins, serpin-like proteins) that function in defense processes through an effect on exogenous proteolytic attack by the pathogens, in plants like sugarcane, poplar, grapevine, potato and kiwi [21,36,37,58,72,77].

Other proteins with stress-antifungal properties detected in stress conditions are the cysteine-rich repeat secretory protein 55-like, also previously detected in coffee [20]. In poplar and Arabidopsis this protein was found together with a cysteine-rich repeat secretory protein 38-like which seems to be structurally similar with a lipid transfer protein from tomato and can, thus, be classified as a PR-14 protein [21,73].

It is evident, from the analysis of the results that PR-proteins have an important contribution to “response to stimulus” even in the non-challenged conditions. This fact is indicative of the existence of a constitutive immune system in the plant ECS which is in accordance with the several previous reports [19,20,64,78,79].

### 3.3. Protein Metabolism

In our review, of the proteins ascribed to protein metabolism the majority are proteases. They are mainly of the serine-, cysteine-, and aspartyl-type, but not threonine- or metallo-proteases. Data available at the MEROPS database [80,81] (Supplementary Table S2) show that, at the genome level, the serine-type proteases are typically the most abundant, followed by the aspartic-type and cysteine-type. We have also detected a higher amount of serine-type proteases (subtilisin and serine carboxy peptidases), followed by aspartic-type proteases and cysteine-type proteases. In the dicot plants, proteolysis seems to be more important for biotic stress and non-challenged conditions than for abiotic stress, for which serine-type proteases, particularly subtilisins, have a major contribution. On the other hand, it seems that abiotic stress does not lead to an investment in proteases other than aspartic-type (in fact, a relative decrease is observed for all the other proteases).

Several cellular roles are described for proteases. In general terms, they could be required to remove signal peptides from secretory proteins; to remove propeptides from proteins that are synthesized as precursors; to release bioactive peptides; to release proteins from the cell surface (shedding); or to inactivate xenoproteins from parasites or pathogens [82]. Some cysteine-type proteases, including those described as being involved in pathogen perception and disease resistance signaling (key components of plant immunity) [83], were shown to increase in the apoplast of a coffee resistant genotype during the early response to leaf rust [20]. However, later on, serine-type proteases (subtilases and serine carboxypeptidases) were the most relevant in this interaction. In grapevine, a subtilisin-like protein sharing sequence similarity with the tomato P69 (PR-7 protein) was shown to be constitutively expressed in the resistant genotype and induced after *Plasmopara viticola* infection [84,85].

It should be noted that according to van der Hoorn [82], the Arabidopsis genome contains many subtilisin and aspartic-type proteases but a biological role is known for only two subtilisin proteases (pattern of stomata development; cuticle development) and for two aspartic-type proteases (disease resistance signaling; cell survival during gametogenesis and embryogenesis). This indicates that the specific role of each protease is still unknown in most cases.

Under the abiotic stress condition three other β-*N*-acetylhexosaminidases (HEXO) proteins were also annotated in protein metabolism, being involved in protein glycosylation. β-*N*-acetylhexosaminidases belong to the GH20 and are involved in the N-glycan processing of secretory glycoproteins when located in the plasma membrane as HEXO2 and HEXO3 [86]. In Arabidopsis, a minor proportion of HEXO3 is found to be soluble in the extracellular space [87]. HEXO proteins were suggested to be involved in plant defense reactions by hydrolyzing *N*-acetylglucose residues from chitin [85] and to be associated with the fruit softening process during ripening [88]. In coffee, leaf apoplastic HEXO proteins changed in abundance with daily temperature amplitude [19].

### 3.4. Redox

It is known that the ECS has a significant contribution to the redox-signaling network in plants [89]. By analyzing the paper dataset of this review, we found that about 8% of proteins are involved in the redox metabolism, independently of the experimental conditions (abiotic, biotic, or non-challenged, Figure 3), and are mostly peroxidases and SOD.

Peroxidases are important oxidoreductase enzymes responsible for the stress-induced formation and degradation of ROS (^•^OH, HOO^•^, H_2_O_2_). They are of significant relevance in both development and response to the environment, being involved in lignification, suberization, auxin catabolism, wound/healing, and defense against pathogens [90]. The major peroxidases present in plant ECS are of class III, for which more than 100 genes were found in plant genomes. By phylogenetic analysis several class III peroxidases can be identified, as well as their promoters [90,91,92,93]. While producing information on specific functions and localizations, it is difficult to define specific functions for each form due to their low substrate specificity in the *in vitro* assays [94]. Therefore, their biological function was mainly defined making use of the characteristics of transgenic plants over- or under-expressing a specific peroxidase isoform. However, it has been proposed that some peroxidases enhance resistance against pathogens through lignification that reinforces the plant cell wall [21,72]. These peroxidases involved in cell wall lignification were classified as PR-9 proteins [64].

The second most represented proteins, the SODs, precede peroxidases in their action in reactive oxygen species (ROS) metabolism, by oxidizing superoxide (O_2_^•−^) to H_2_O_2_ and oxygen. The germin-like proteins (GLPs), already referred in the *Response to Stimulus* section can also have SOD activity. All types of SODs with Cu-Zn, Mn, or Fe co-factors were already found in the extracellular space of plants [95]. SODs that have been detected during pathogen response, together with peroxidases are the first line of defense against ROS [20,96,97]. The activities of the oxi-reductase observed during pathogen response indicates that plants were either initiating the production of ROS to directly fight the pathogen or responding to oxidative intermediates produced as a result of cell wall or membrane damage leading to cell death during HR response [59,78,96]. The ROS metabolism, together with the auxin balance, control the cell wall remodeling (loosening or strengthening) during development and stress response [21,72].

### 3.5. Signaling

Less than 6% of proteins found in the APF of this review are involved in signaling (Figure 3). This is not surprising since we can envisage difficulties in detecting molecules that participate in signaling. For instance, it is known that there are APF signaling peptides (e.g., systemin, phytosulphokine, CLAVATA3) [98,99] that, due to their small size, are not detected by the current 2-DE techniques. Proteases are important in this context since they can proteolytically process some signaling effectors and also lead to the formation of small peptides [99]. On the other hand, some proteins involved in these processes have extracellular domains covalently bound to the plasma membrane or are transmembrane proteins. Proteins with kinase activity are the most represented in this category followed by proteins with the LRR-domain and the phosphorylated extracellular-like proteins.

Several groups or families of kinases are present in plant genomes and are involved in a wide range of functions, from cell signaling to metabolism. Some of these kind of proteins found in our APF dataset are serine-threonine protein kinase, leucine-rich repeat receptor kinase (LRR-receptor kinase), CLAVATA1, DUF26, EP1/AED19, Ca/calmodulin-dependent protein kinase, and mitogen-activated protein kinase. Proteins with a LRR-domain, such as lectins-like proteins, were found in the APF of Arabidopsis and potato [12,36,39,57,100]. The LRR domain (a sequence of 20–29 amino acid residues) is a versatile structure that favors the formation of protein-protein and/or -polysaccharide interactions. LRR proteins are numerous and include the, LRR-containing receptor-like kinases (LRR-RLKs), LRR-containing receptor-like proteins (LRR-RLPs) and polygalacturonase inhibitor proteins (PGIPs) and have diverse functions [101] with a prominent role in plant defense, among other biological functions. “They can provide an early warning system for the presence of potential pathogens and activate protective immune signaling in plants. In addition, they act as a signal amplifier in the case of tissue damage, establishing symbiotic relationships and affecting developmental processes” [102].

Some of the GHs detected in this review can also participate in signaling, such as the heparanase-like proteins of plants that have β-glucuronidase (GUS) activity and are placed in the GH 79 family. These extracellular enzymes are shown to act on AGPs [103] and, thus, having a role in the regulation of cell growth. The heparanase found in poplar and maize APFs is indirectly involved in H_2_O_2_ degradation [21,104]. At the same time, it generates phenolic compounds that may be used for cell wall fortification [72].

The enzyme invertase was also detected. It has been shown that this enzyme, by itself, or in combination with plant hormones, can regulate many aspects of growth and development of plants, because its substrate and products are not only nutrients, but also signal molecules [105]. Concerning cell wall invertase, it was further indicated that it regulates source/sink carbohydrate partitioning in plants and also has a role of PR-protein [106].

These results give evidence on a number of signaling processes that are taking place in the ECS. To the proteins here described others also can be added, though included in other sections of the review because they have multifunctional roles, like those ascribed to “*miscellaneous enzyme families*” or that participate in “redox” processes.

### 3.6. Other Metabolic Processes

Several proteins involved in *secondary metabolism* were also detected in the APF of the three experimental conditions. The most abundant ones were the reticuline-like oxidases (berberine bridge enzyme-like and reticuline oxidase) and cinnamyl-alcohol dehydrogenase. Reticuline-like oxidases (FAD-family) are monolignol oxidoreductases that play a role in monolignol metabolism and lignin formation [73,107]. These enzymes may participate in the mobilization and oxidation of monolignols (aromatic allylic alcohols, such as coumaryl-, sinapyl-, and coniferyl alcohol), to the corresponding aldehydes. Both monolignols and their aldehydes can be secreted into the apoplast and polymerized to various cell wall components (e.g., lignification) [20,107]. In Arabidopsis, it seems that reticulin-like oxidases and some GHs work in concert, being involved in the formation of cell wall components required during certain growth phases and in response to stress. Furthermore, the presence of homologs of Arabidopsis berberine bridge enzyme in other plants indicates a general role of this enzyme family in the plant kingdom [107]. The cinnamyl-alcohol dehydrogenase enzyme (CAD), which participates in one of the last steps of biosynthesis of monolignols, prior to polymerization into the lignin polymer, is important in providing structural support, hydrophobicity, and protection against pathogens [108].

At a lower frequency some proteins/enzymes are functionally categorized as *miscellaneous enzyme families*. As reported by Albenne et al. [109] this class is the “Achilles' heel of the classification since it comprises all the proteins which cannot be put elsewhere”. These enzymes are multi-functional, active in many metabolic pathways and different stress conditions [109]. In this review, this class is mostly represented by GDSL-like lipases (Gly-Asp-Ser-(Leu) motif with a N-terminus active serine residue) having a vital role in development and defense [110], and by the metallophosphatases purple acid phosphatase and calcineurin-like phosphoesterase, involved in the generation of reactive oxygen species, flower development, and cell wall biosynthesis [20,111]. We have identified a GDSL-motif lipase/hydrolase and calcineurin-like phosphoesterase (a calcium–dependent phosphatase) in the early phase of the resistant response of coffee to *H. vastatrix*, what suggests the potential involvement of these proteins in pathogen perception and signal transduction cascades [20]. In *Arabidopsis thaliana* a GDSL LIPASE1 protein seems to protect plants from *Alternaria brassicicola* attack in two distinct ways: by directly disrupting fungal spore integrity, and by activating defense signaling in plants [112]. It is known that upon perception of microbial signals, kinases, and phosphatases target specific proteins, often modifying complex signaling cascades that allow for rapid defense responses [79]. The presence of phosphatases in the extracellular proteome of Arabidopsis infected with *Pseudomonas syringae* suggests that potential phosphorylation/dephosphorylation reversible regulation could occur in the apoplast [113].

Aldose-epimerase proteins were categorized in *minor carbohydrate metabolism*, with a molecular function of carbohydrate-binding or isomerase activity. They can be involved in the formation of carbohydrate derivatives by the addition of a carbohydrate residue to another molecule. They interact selectively and non-covalently with any carbohydrate, which includes monosaccharides, oligosaccharides, and polysaccharides, as well as substances derived from monosaccharides by reduction of the carbonyl group (alditols), by oxidation of one or more hydroxy groups to afford the corresponding aldehydes, ketones, or carboxylic acids, or by replacement of one or more hydroxy group(s) by a hydrogen atom (UniProtKB database). They have been detected in ECS of various plants and can be involved either on the cell wall reinforcement/organization or in cell wall biogenesis [19,20,73,101,114].

## 4. Conclusions and Future Perspectives

Apoplastic proteomics provides a global view of the ECS proteome and enables the study of changes that occur as a result of development or in relation to abiotic or biotic environmental responses. Despite technical limitations still persisting, recent improvements of the technologies for separation and identification of APF proteins have contributed to a better understanding of the ECS proteome. The present review is focused on 2003–2015 ECS proteomic papers and intends to draw information on the metabolic processes occurring in the ECS under abiotic and biotic stresses and non-challenged conditions. However, the reduced number of papers on monots and on some plant organs only allowed extracting useful information for leaves of dicots.

The main biological processes detected in the ECS are “cell wall organization and biogenesis”, “response to stimulus” and “protein metabolism”. When comparing the three different experimental conditions, it is seen that the same types of proteins are found for each of them, but with differences on their relative contribution. So, for both stress conditions “response to stimulus” was the most represented biological process, while “cell wall organization and biogenesis” was more represented in the non-challenged plants. For the stress studies it appears that “protein metabolism” is more represented in the biotic stress than in the abiotic stress. The fact that in the non-challenged plants the detected proteins are identical to those of the stress situations suggests that non-challenged plants have intrinsic constitutive metabolism processes of stress/defense.

A large number of proteins present in the apoplast are enzymes or proteins involved in processes related with the cell wall (structure, remodeling, signaling, and defense). Additionally, the proteins that were categorized in “cell wall organization or biogenesis” several others ascribed to a different biological process also have implications in cell wall structure or remodeling. The major enzymes responsible for cell wall polysaccharide breakdown and modification are GHs and carbohydrate esterases. Other GHs function as PR-like proteins (chitinases, glucanases, and thaumatin-like) being involved in stress/defense responses. Identically, germin-like proteins, highly detected in the APF, can be considered PR-proteins although also participating in redox metabolism. Equally important are the peroxidases and SODs that are frequently implicated in the generation of ROS, lignification, and defense against pathogens. Furthermore, ROS can also act as signal molecules leading to the activation of kinases and phosphatases, often modifying complex signal cascades that differentially regulated genes. This analysis shows that cell wall structure modulation is a permanent process in the cell, fundamental for its survival under changing environments. Proteases can also contribute to the complex processes that are going on in the ECS by means of their role in the maturation of enzymes, signaling, protein turnover, and defense.

These observations should be taken critically, not only because of the limited amount of information available but, most importantly, also for the difficulty in ascribing to each detected protein a specific function in a particular biological process. Indeed, many proteins have multiple functions and a biological process like “signaling” cannot be considered independently without its connections to “redox processes” and “responses to stimulus”.

It is evident that the biological processes taking place in the ECS are very complex and the proteome should be considered in its complexity and as a whole. In addition to the multiple functions ascribed to the ECS proteins, it should not be forgotten that they interact between themselves and with the plasma membrane and its components. These interactions are crucial in connecting exterior and interior of the cell, and even simple protein actions in the ECS can have profound effects on plant behavior. So, the several ECS metabolic molecules appear to be permanently contributing to the high dynamic nature of this plant compartment.

Futures perspectives for improving the knowledge on the APF proteins:

- Increase the number of available publications with access to quantitative data, to have a better picture of the plant ECS both in commelinid monocots and dicot plants and in different tissues/organs.
- Despite all of the improvements of 2-DE associated with MS, technical limitations still persist for the detection of certain types of polypeptides. For instance, small apoplastic peptides which have fundamental signaling roles are lost during the common procedures and their detection requires a special 2-DE methodology [115]. Furthermore, woody plants (grapevine, poplar, coffee) present additional problems due to the difficulty in extracting and separating their proteins. Solubilization of the ECS proteins is also a matter of concern since these proteins could be tightly trapped into the extracellular matrix or be adsorbed to cell wall components. Specialist approaches of cell wall treatment could be attempted to solve the problem. On the other hand the use of fractionation techniques of the APF (e.g., by means of affinity columns) could provide additional information on certain types of proteins, isoform level, and regulatory components. Some of these problems of protein separation and analysis can be solved if high-throughput gel-free techniques can be used, which normally implies the availability of complete sequenced genomes.
- Another important point that needs improvement is the validation of the sub-cellular localization of the proteins. The eventual presence of cytoplasmic proteins in the ECS is a situation that should be dealt with care and technical precautions should be taken to reduce possible contamination. However, the presence of the leaderless or non-classically secreted proteins in the apoplast cannot be discarded. For instance, accumulated evidence from several plant species suggests the existence of exosome-like structures that carry and deliver specific proteins to the ECS, with still-undiscovered functions [27,116,117].
- Information stored on MS databases should be improved since proteins are sometimes difficult to identify. Examples of the problems encountered are the heterogeneity in biological function annotation that is a consequence of the complex cellular roles of the proteins; the poor representation of proteins less abundant in the cells; and the frequent lack of information on PTMs, such as glycosylation.
- In order to fully understand all the complexity of the processes that involve the extracellular proteins it would be necessary to build networks of interactions between the diversity of those proteins.

In conclusion, by using proteomic analysis it was possible to start to have a global picture of the different families of proteins present in the ECS, their relationships, and how they perceive the environment and orchestrate a molecular response. These data opened new working hypotheses for this complex network of molecules that form the ECS.

## Supplementary Materials

The following are available online at www.mdpi.com/2227-7382/4/3/22/s1, Table S1: Original research articles in plant apoplastic proteins. The bibliographic search on the Web of Knowledge (http://apps.webofknowledge.com, 1 December 2015) was made using as search topic “plant apoplastic proteom*”. Table S2: Known and putative proteases available at the Merops database (accessed on 12 March 2016).
