# Supplementary material for: Protein Dynamics in the Plant Extracellular Space
Source: Proteomes. 2016 Jul 13;4(3):22. doi: 10.3390/proteomes4030022 (PMC5217353; doi:10.3390/proteomes4030022)
Supplement: Supplementary file 1 [file proteomes-04-00022-s001.pdf]

Protein Dynamics in the Plant Extracellular Space

Leonor Guerra-Guimarães, Carla Pinheiro, Inês Chaves, Danielle R. Barros and Cândido P. Ricardo

Table S1. Original research articles in plant apoplastic proteins. The bibliographic search on the Web of Knowledge (http://apps.webofknowledge.com, 1st December 2015) was made using as search topic "plant apoplastic proteome*".

Reference	Plant	Treatment	Plant organ	Separation Method	Year of Publication
[1]	*Solanum tuberosum*	*Phytophthora infestans*	leaf	gel based (1D)	2014
[2]	*Lupinus albus*	boron deficiency	leaf	gel based (2D)	2006
[3]	*Triticum aestivum*	*Zymoseptoria tritici*	leaf	gel based (1D) & gel free (LC)	2015
[4]	*Solanum tuberosum*	BABA 1 e 10mM + *Phytophthora infestans*	leaf	gel based (1D) followed by LC	2014
[5]	*Arabidopsis thaliana*	Methods (infiltration solutions)	rosettes	gel based (2D)	2005
[6]	*Arabidopsis thaliana*	*Pseudomonas syringae*	leaf	gel based (2D) & gel free (ICPL-LC)	2014
[7]	*Prunus persica*	post-harvest heat treatment	fruit mesocarp	gel based (2-DIGE)	2012
[8]	*Arabidopsis thaliana*	oligogalacturonides	seedling	gel based (2-DIGE) & gel free (LC)	2008
[9]	*Beta vulgaris*	Fe deficiency and Fe resupply	leaf	gel based (2D) & gel free (LC)	2015
[10]	*Solanum lycopersicum*	*Ralstonia solanacearum*	stem	gel based (2D) & 2D followed by LC	2010
[11]	*Nicotiana tabacum*	salt	leaf	gel based (2D) followed by LC	2005
[12]	*Linum usitatissimum*	cell wall study	stem	gel based (2D) followed by LC	2013
[13]	*Vitis vinifera*	Large-scale proteomic analysis	leaf	gel based (2D)	2013
[14]	*Prunus persica*	plum pox virus (PPV)	leaf	gel based (2D)	2006
[15]	*Glycine max*	symbiosis (*Bradyrhizobium japonicum*)	Hypocotyl, epicotyl and stem tissue	gel based (1D & 2D) followed by LC	2007
[16]	*Vigna unguiculata*	manganese toxicity	leaf	gel based (2D) followed by LC	2003

Table S1. *Cont.*

Reference	*Plant*	Treatment	Plant organ	Separation Method	Year of Publication
[17]	*Solanum tuberosum*	wounding and *Phytophthora infestans*	tubers	gel based (1D)	2012
[18]	*Brassica oleracea*	salt	xylem sap	gel free (LC)	2011
[19]	*Arabidopsis thaliana*	*Verticillium longisporum*	leaf	gel based (2D) followed by LC	2012
[20]	*Oryza sativa*	manganese sensitivity and leaf age	leaf	gel based (2D)	2010
[21]	*Vigna unguiculata*	toxic manganese supply and silicon	leaf	gel based (1D) followed by LC	2009
[22]	*Nicotiana benthamiana*	DataSet brief (2-DE proteome map)	leaf	gel based (2D)	2010
[23]	*Coffea arabica*	*Hemileia vastatrix*	leaf	gel based (2D)	2015
[24]	*Coffea arabica*	Temperature amplitude and non-challenged	leaf	gel based (2D)	2014
[25]	*Coffea arabica*	*Hemileia vastatrix*	leaf	gel based (2-DIGE)	2012
[26]	*Arabidopsis + rice + triticum aestivum*	enriched apoplastic extracts	leaf	gel based (2D)	2003
[27]	*Nicotiana tabacum*	phosphoproteomic analysis	culture cells	gel based (2D)	2010
[28]	*Oryza sativa*	*Cochliobolus miyabeanus*	leaf	gel based (2D)	2014
[29]	*Oryza sativa*	*Magnaporthe oryzae*	leaf	gel based (2-DIGE) & gel free (LC)	2013
[30]	*Arabidopsis thaliana*	cell wall regeneration	protoplasts of suspension-culture d cells	gel based (2D)	2005
[31]	*Brassica oleracea*	study of the proteome	xylem sap	gel based (1D) followed by LC	2011
[32]	*Arabidopsis thaliana*	salicylic acid and *Alternaria brassicicola*	culture cells	gel based (2D)	2005
[33]	*Oryza sativa*	Dehydration	seedlings	gel based (2D) followed by LC	2010
[34]	*Populus spp.*	Apoplast proteome	leaf and stem	gel based (2D) followed or not by LC	2010
[35]	*Actinidia deliciosa*	*Pseudomonas syringae*	leaf	gel based (2D) followed by LC	2014

Table S1. *Cont.*

Reference	*Plant*	Treatment	Plant organ	Separation Method	Year of Publication
[36]	*Hordeum vulgare*	Cadmium Stress	leaf	gel based (1D) followed by LC and gel based (2D)	2011
[37]	*Brassica juncea*	NO + cold stress signaling	seedlings	gel based (1D & 2D) followed by LC	2014
[38]	*Oryza sativa*	*Magnaporthe sp*	leaf	gel based (2D) followed by LC	2012
[39]	*Medicago truncatula*	wound	leaf	gel based (2D)	2009
[40]	*Oryza sativa*	NaCl	shoot stem	gel based (2-DIGE) followed by LC	2011
[41]	*Arabidopsis thaliana*	Ascorbate deficiency	leaf cell wall proteome	gel free (LC)	2015
[42]	*Nicotiana tabacum*	flagellin treated	leaf	gel based (2D) followed by LC	2012
[43]	*Arabidopsis thaliana*	UV-B radiation + oxidative stress	leaf	gel based (1D) followed by LC	2015
[44]	*Arabidopsis thaliana*	natriuretic peptides (PNPs)	cell suspention cultures	gelfree (offgel fraccionator)	2014
[45]	*Arabidopsis thaliana*	germinated vs ungerminated	pollen	gel based (2-DIGE) followed by LC	2011
[46]	*Pisum sativum*	*Nectria haematococca*	root tip	gel free (LC)	2006
[47]	*Zea mays*	Methods (infiltration solutions)	leaf	gel based (1D & 2D) and gel free (LC)	2011
[48]	*Oryza sativa*	*Magnaporthe oryzae*	leaf	gel based (2D)	2014
[49]	*Triticum aestivum*	*Zymoseptoria tritici*	leaf	gel free (iTRAQ)	2015
[50]	*Phaseolus vulgaris*	polyethylene glycol-induced osmotic stress	root tip	gel based (2D) followed by LC	2013
[51]	*Oryza sativa*	salt NaCl	root	gel based (2D)	2009
[52]	*Oryza sativa*	hydrogen peroxide	seedling root	gel based (2D)	2011

Table S2. Known and putative proteases available at the Merops database (accessed on 12 March 2016) [53,54].

	Athaliana	*Carabica*	*Ccanephora*	*Osativa*	*Zmays*
Nº known & putative peptidases	789	5	414	1427	1051
Nº families	60	4	51	65	53
Nº clans	29	4	26	31	31
Cysteine-type					
Nº families	19	2	16	21	20
Nº peptidases	165	2	85	286	285
% of the total	21%	40%	21%	20%	27%
Serine-type					
Nº families	14	3	12	15	15
Nº peptidases	301	3	183	373	395
% of the total	38%	60%	44%	26%	38%
Aspartic-type					
Nº families	4	-	4	5	4
Nº peptidases	187	-	57	595	193
% of the total	24%	-	14%	42%	18%
others					
Nº families	23	-	19	24	24
Nº peptidases	136	-	89	173	178
% of the total	17%		21%	12%	17%

Non-peptidase homologues were not considered (n=98 for At; n=346 for Os; n=72 for Cc; n=239 for Zm).

References

1. Ali, A.; Alexandersson, E.; Sandin, M.; Resjö, S.; Lenman, M.; Hedley, P.; Levander, F.; Andreasson, E. Quantitative proteomics and transcriptomics of potato in response to *Phytophthora infestans* in compatible and incompatible interactions. *BMC Genomics* **2014**, *15*, 497.
2. Alves, M.; Francisco, R.; Martins, I.; Ricardo, C.P.P. Analysis of *Lupinus albus* leaf apoplastic proteins in response to boron deficiency. *Plant Soil* **2006**, *279*, 1–11.
3. Ben M'Barek, S.; Cordewener, J.H.G.; van der Lee, T.A.J.; America, A.H.P.; Mirzadi Gohari, A.; Mehrabi, R.; Hamza, S.; de Wit, P.J.G.M.; Kema, G.H.J. Proteome catalog of *Zymoseptoria tritici* captured during pathogenesis in wheat. *Fungal Genet. Biol.* **2015**, *79*, 42–53.
4. Bengtsson, T.; Weighill, D.; Proux-Wéra, E.; Levander, F.; Resjö, S.; Burra, D.D.; Moushib, L.I.; Hedley, P.E.; Liljeroth, E.; Jacobson, D.; et al. Proteomics and transcriptomics of the BABA-induced resistance response in potato using a novel functional annotation approach. *BMC Genomics* **2014**, *15*, 315.
5. Boudart, G.; Jamet, E.; Rossignol, M.; Lafitte, C.; Borderies, G.; Jauneau, A.; Esquerré-Tugayé, M.-T.; Pont-Lezica, R. Cell wall proteins in apoplastic fluids of *Arabidopsis thaliana* rosettes: identification by mass spectrometry and bioinformatics. *Proteomics* **2005**, *5*, 212–221.
6. Breitenbach, H.H.; Wenig, M.; Wittek, F.; Jordá, L.; Maldonado-Alconada, A.M.; Sarioglu, H.; Colby, T.; Knappe, C.; Bichlmeier, M.; Pabst, E.; et al. Contrasting Roles of the Apoplastic Aspartyl Protease APOPLASTIC, ENHANCED DISEASE SUSCEPTIBILITY1-DEPENDENT1 and LEGUME LECTIN-LIKE PROTEIN1 in Arabidopsis Systemic Acquired Resistance. *Plant Physiol.* **2014**, *165*, 791–809.
7. Bustamante, C.; Budde, C.O.; Borsani, J.; Lombardo, V.; Lauxmann, M.; Andreo, C.S.; Lara, M.V.; Drincovich, M.F. Heat treatment of peach fruit: modifications in the extracellular compartment and identification of novel extracellular proteins. *Plant Physiol. Biochem.* **2012**, *60*, 35–45.
8. Casasoli, M.; Spadoni, S.; Lilley, K.S.; Cervone, F.; De Lorenzo, G.; Mattei, B. Identification by 2-D DIGE of apoplastic proteins regulated by oligogalacturonides in *Arabidopsis thaliana*. *Proteomics* **2008**, *8*, 1042–1054.
9. Ceballos-Laita, L.; Gutierrez-Carbonell, E.; Lattanzio, G.; Vázquez, S.; Contreras-Moreira, B.; Abadía, A.; Abadía, J.; López-Millán, A.-F. Protein profile of *Beta vulgaris* leaf apoplastic fluid and changes induced by Fe deficiency and Fe resupply. *Front. Plant Sci.* **2015**, *6*, 145.

10. Dahal, D.; Pich, A.; Braun, H.P.; Wydra, K. Analysis of cell wall proteins regulated in stem of susceptible and resistant tomato species after inoculation with *Ralstonia solanacearum*: A proteomic approach. *Plant Mol. Biol*. **2010**, *73*, 643–658.
11. Dani, V.; Simon, W.J.; Duranti, M.; Croy, R.R.D. Changes in the tobacco leaf apoplast proteome in response to salt stress. *Proteomics* **2005**, *5*, 737–745.
12. Day, A.; Fénart, S.; Neutelings, G.; Hawkins, S.; Rolando, C.; Tokarski, C. Identification of cell wall proteins in the flax (*Linum usitatissimum*) stem. *Proteomics* **2013**, *13*, 812–825.
13. Delaunois, B.; Colby, T.; Belloy, N.; Conreux, A.; Harzen, A.; Baillieul, F.; Clément, C.; Schmidt, J.; Jeandet, P.; Cordelier, S. Large-scale proteomic analysis of the grapevine leaf apoplastic fluid reveals mainly stress-related proteins and cell wall modifying enzymes. *BMC Plant Biol*. **2013**, *13*, 24.
14. Diaz-Vivancos, P.; Rubio, M.; Mesonero, V.; Periago, P.M.; Ros Barceló, A.; Martínez-Gómez, P.; Hernández, J.A. The apoplastic antioxidant system in Prunus: Response to long-term plum pox virus infection. *J. Exp. Bot.* **2006**, *57*, 3813–3824.
15. Djordjevic, M.A.; Oakes, M.; Li, D.X.; Hwang, C.H.; Hocart, C.H.; Gresshoff, P.M. The Glycine max Xylem Sap and Apoplast Proteome. *J. Proteome Res*. **2007**, *6*, 3771–3779.
16. Fecht-Christoffers, M.M.; Braun, H.-P.; Lemaitre-Guillier, C.; VanDorsselaer, A.; Horst, W.J. Effect of manganese toxicity on the proteome of the leaf apoplast in cowpea. *Plant Physiol*. **2003**, *133*, 1935–1946.
17. Fernández, M.B.; Pagano, M.R.; Daleo, G.R.; Guevara, M.G. Hydrophobic proteins secreted into the apoplast may contribute to resistance against *Phytophthora infestans* in potato. *Plant Physiol. Biochem*. **2012**, *60*, 59–66.
18. Fernandez-Garcia, N.; Hernandez, M.; Casado-Vela, J.; Bru, R.; Elortza, F.; Hedden, P.; Olmos, E. Changes to the proteome and targeted metabolites of xylem sap in *Brassica oleracea* in response to salt stress. *Plant Cell Environ*. **2011**, *34*, 821–836.
19. Floerl, S.; Majcherczyk, A.; Possienke, M.; Feussner, K.; Tappe, H.; Gatz, C.; Feussner, I.; Kües, U.; Polle, A. *Verticillium longisporum* infection affects the leaf apoplastic proteome, metabolome, and cell wall properties in *Arabidopsis thaliana*. *PLoS ONE* **2012**, 7, e31435.
20. Fuhrs, H.; Behrens, C.; Gallien, S.; Heintz, D.; van Dorsselaer, A.; Braun, H.-P.; Horst, W.J. Physiological and proteomic characterization of manganese sensitivity and tolerance in rice (*Oryza sativa*) in comparison with barley (*Hordeum vulgare*). *Ann. Bot*. **2010**, *105*, 1129–1140.
21. Fuhrs, H.; Gotze, S.; Specht, A.; Erban, A.; Gallien, S.; Heintz, D.; van Dorsselaer, A.; Kopka, J.; Braun, H.-P.; Horst, W.J. Characterization of leaf apoplastic peroxidases and metabolites in *Vigna unguiculata* in response to toxic manganese supply and silicon. *J. Exp. Bot.* **2009**, *60*, 1663–1678.
22. Goulet, C.; Goulet, C.; Goulet, M.; Michaud, D. 2-DE proteome maps for the leaf apoplast of *Nicotiana benthamiana*. *Proteomics* **2010**, *10*, 2536–2544.
23. Guerra-Guimarães, L.; Tenente, R.; Pinheiro, C.; Chaves, I.; Silva, M.D.C.; Cardoso, F.M.H.; Planchon, S.; Barros, D.R.; Renaut, J.; Ricardo, C.P. Proteomic analysis of apoplastic fluid of *Coffea arabica* leaves highlights novel biomarkers for resistance against *Hemileia vastatrix*. *Front. Plant Sci*. **2015**, *6*, 1–16.
24. Guerra-Guimarães, L.; Vieira, A.; Chaves, I.; Pinheiro, C.; Queiroz, V.; Renaut, J.; Ricardo, C.P. Effect of greenhouse conditions on the leaf apoplastic proteome of *Coffea arabica* plants. *J. Proteomics* **2014**, *104*, 128–139.
25. Guerra-Guimarães, L.; Vieira, A.; Chaves, I.; Queiroz, V.; Pinheiro, C.; Renaut, J.; Silva, L.; Zambolim, L.; Ricardo, C.; Silva, M.C. Cytologic and Proteomic Analysis of *Coffea arabica–Hemileia vastatrix* Interactions. Proceedings of 24th International Conference on Coffee Science (ASIC), San Jose, CA, USA, 11–16 November 2012; pp. 1414–1418.
26. Haslam, R.P.; Downie, A.L.; Raveton, M.; Gallardo, K.; Job, D.; Pallett, K.E.; John, P.; Parry, M.A.J.; Coleman, J.O.D. The assessment of enriched apoplastic extracts using proteomic approaches. *Annals of Applied Biology* **2003**, *143*, 81–91.
27. Kaida, R.; Serada, S.; Norioka, N.; Norioka, S.; Neumetzler, L.; Pauly, M.; Sampedro, J.; Zarra, I.; Hayashi, T.; Kaneko, T.S. Potential role for purple acid phosphatase in the dephosphorylation of wall proteins in tobacco cells. *Plant Physiol*. **2010**, *153*, 603–610.
28. Kim, J.Y.; Wu, J.; Kwon, S.J.; Oh, H.; Lee, S.E.; Kim, S.G.; Wang, Y.; Agrawal, G.K.; Rakwal, R.; Kang, K.Y.; et al. Proteomics of rice and *Cochliobolus miyabeanus* fungal interaction: Insight into proteins at intracellular and extracellular spaces. *Proteomics* **2014**, *14*, 2307–2318.

29. Kim, S.G.; Wang, Y.; Lee, K.H.; Park, Z.Y.; Park, J.; Wu, J.; Kwon, S.J.; Lee, Y.H.; Agrawal, G.K.; Rakwal, R.; et al. In-depth insight into *in vivo* apoplastic secretome of rice-*Magnaporthe oryzae* interaction. *J. Proteomics* **2013**, *78*, 58–71.
30. Kwon, H.K.; Yokoyama, R.; Nishitani, K. A proteomic approach to apoplastic proteins involved in cell wall regeneration in protoplasts of Arabidopsis suspension-cultured cells. *Plant Cell Physiol*. **2005**, *46*, 843–857.
31. Ligat, L.; Lauber, E.; Albenne, C.; San Clemente, H.; Valot, B.; Zivy, M.; Pont-Lezica, R.; Arlat, M.; Jamet, E. Analysis of the xylem sap proteome of *Brassica oleracea* reveals a high content in secreted proteins. *Proteomics* **2011**, *11*, 1798–813.
32. Oh, I.S.; Park, A.R.; Bae, M.S.; Kwon, S.J.; Kim, Y.S.; Lee, J.E.; Kang, N.Y.; Lee, S.; Cheong, H.; Park, O.K. Secretome analysis reveals an Arabidopsis lipase involved in defense against *Alternaria brassicicola*. *Plant Cell* **2005**, *17*, 2832–2847.
33. Pandey, A.; Rajamani, U.; Verma, J.; Subba, P.; Datta, A.; Chakraborty, S.; Chakraborty, N.; Accepted, J. Identification of Extracellular Matrix Proteins of Rice (*Oryza sativa* L.) Involved in Dehydration-Responsive Network : A Proteomic Approach. *J. Proteome Res*. **2010**, *9*, 3443–3464.
34. Pechanova, O.; Hsu, C.-Y.; Adams, J.P.; Pechan, T.; Vandervelde, L.; Drnevich, J.; Jawdy, S.; Adeli, A.; Suttle, J.C.; Lawrence, A.M.; et al. Apoplast proteome reveals that extracellular matrix contributes to multistress response in poplar. *BMC Genomics* **2010**, *11*, 674.
35. Petriccione, M.; Salzano, A.M.; di Cecco, I.; Scaloni, A.; Scortichini, M. Proteomic analysis of the *Actinidia deliciosa* leaf apoplast during biotrophic colonization by *Pseudomonas syringae* pv. *actinidiae*. *J. Proteomics* **2014**, *101*, 43–62.
36. Pos, V.; Hunyadi-Gulyas, E.; Caiazzo, R.; Jocsak, I.; Medzihradszky, K.F.; Lukacs, N. Induction of Pathogenesis-Related Proteins in Intercellular Fluid by Cadmium Stress in Barley (*Hordeum vulgare* L.)—A Proteomic Analysis. *Acta Aliment*. **2011**, *40*, 164–175.
37. Sehrawat, A.; Deswal, R. *S*-nitrosylation analysis in *Brassica juncea* apoplast highlights the importance of nitric oxide in cold-stress signaling. *J. Proteome Res*. **2014**, *13*, 2599–619.
38. Shenton, M.R.; Berberich, T.; Kamo, M.; Yamashita, T.; Taira, H.; Terauchi, R. Use of intercellular washing fluid to investigate the secreted proteome of the rice-Magnaporthe interaction. *J. Plant Res*. **2012**, *125*, 311–316.
39. Soares, N.C.; Francisco, R.; Ricardo, C.P.; Jackson, P.A. Proteomics of ionically bound and soluble extracellular proteins in *Medicago truncatula* leaves. *Proteomics* **2007**, *7*, 2070–2082
40. Song, Y.; Zhang, C.; Ge, W.; Zhang, Y.; Burlingame, A.L.; Guo, Y. Identification of NaCl stress-responsive apoplastic proteins in rice shoot stems by 2D-DIGE. *J. Proteomics* **2011**, *74*, 1045–1067.
41. Sultana, N.; Florance, H.V.; Johns, A.; Smirnoff, N. Ascorbate deficiency influences the leaf cell wall glycoproteome in *Arabidopsis thaliana*. *Plant. Cell Environ*. **2015**, *38*, 375–384.
42. Szabó, E.; Szatmári, Á.; Hunyadi-Gulyás, É.; Besenyei, E.; Zsiros, L.R.; Bozsó, Z.; Ott, P.G. Changes in apoplast protein pattern suggest an early role of cell wall structure remodelling in flagellin-triggered basal immunity. *Biol. Plant*. **2012**, *56*, 551–559.
43. Trentin, A.R.; Pivato, M.; Mehdi, S.M. M.; Barnabas, L.E.; Giaretta, S.; Fabrega-Prats, M.; Prasad, D.; Arrigoni, G.; Masi, A. Proteome readjustments in the apoplastic space of *Arabidopsis thaliana* ggt1 mutant leaves exposed to UV-B radiation. *Front. Plant Sci*. **2015**, *6*, 1–12.
44. Turek, I.; Marondedze, C.; Wheeler, J.I.; Gehring, C.; Irving, H.R. Plant natriuretic peptides induce proteins diagnostic for an adaptive response to stress. *Front. Plant Sci.* **2014**, *5*, 661.
45. Ge, W.; Song, Y.; Zhang, C.; Zhang, Y.; Burlingame, A.L.; Guo, Y. Proteomic analyses of apoplastic proteins from germinating *Arabidopsis thaliana* pollen. *Biochim. Biophys. Acta*. **2011**, *1814*, 1964–1973.
46. Wen, F.; VanEtten, H.D.; Tsaprailis, G.; Hawes, M.C. Extracellular proteins in pea root tip and border cell exudates. *Plant Physiol*. **2007**, *143*, 773–783
47. Witzel, K.; Shahzad, M.; Matros, A.; Mock, H.-P.; Mühling, K.H. Comparative evaluation of extraction methods for apoplastic proteins from maize leaves. *Plant Methods* **2011**, *7*, 48.
48. Wu, J.; Lee, D.Y.; Wang, Y.; Kim, S.T.; Baek, S.-B.; Kim, S.G.; Kang, K.Y. Protein profiles secreted from phylloplane of rice leaves free from cytosolic proteins: Application to study rice-*Magnaporthe oryzae* interactions. *Physiol. Mol. Plant Pathol*. **2014**, *88*, 28–35.

49. Yang, F.; Li, W.; Derbyshire, M.; Larsen, M.R.; Rudd, J.J.; Palmisano, G. Unraveling incompatibility between wheat and the fungal pathogen *Zymoseptoria tritici* through apoplastic proteomics. *BMC Genomics* **2015**, *16*, 362.
50. Yang, Z.-B.; Eticha, D.; Fuhrs, H.; Heintz, D.; Ayoub, D.; van Dorsselaer, A.; Schlingmann, B.; Rao, I.M.; Braun, H.-P.; Horst, W.J. Proteomic and phosphoproteomic analysis of polyethylene glycol-induced osmotic stress in root tips of common bean (*Phaseolus vulgaris* L.). *J. Exp. Bot.* **2013**, *64*, 5569–5586.
51. Zhang, L.; Tian, L.-H.; Zhao, J.-F.; Song, Y.; Zhang, C.-J.; Guo, Y. Identification of an apoplastic protein involved in the initial phase of salt stress response in rice root by two-dimensional electrophoresis. *Plant Physiol*. **2009**, *149*, 916–928.
52. Zhou, L.; Bokhari, S.A.; Dong, C.-J.; Liu, J.-Y. Comparative proteomics analysis of the root apoplasts of rice seedlings in response to hydrogen peroxide. *PLoS ONE* **2011**, 6, e16723.
53. Rawlings, N.D.; Barrett, A.J.; Finn, R. Twenty years of the MEROPS database of proteolytic enzymes, their substrates and inhibitors. *Nucleic Acids Res.* **2016**, *44*, D343–D350.
54. Merops database. Available online: https://merops.sanger.ac.uk (accessed on 12 March 2016)

© 2016 by the authors. Submitted for possible open access publication under the terms and conditions of the Creative Commons Attribution (CC-BY) license (http://creativecommons.org/licenses/by/4.0/).
